# Inhibition of Schwann cell pannexin 1 attenuates neuropathic pain through the suppression of inflammatory responses

**DOI:** 10.1186/s12974-022-02603-x

**Published:** 2022-10-04

**Authors:** Qian Wang, Han-yang Li, Zhuo-min Ling, Gang Chen, Zhong-Ya Wei

**Affiliations:** 1 Key Laboratory of Neuroregeneration of Jiangsu and Ministry of Education, Jiangsu Clinical Medicine Center of Tissue Engineering and Nerve Injury Repair, Co-Innovation Center of Neuroregeneration, Nantong University, Nantong, China; 2 Medical School of Nantong University, Nantong, 226001 Jiangsu China; 3 Department of Anesthesiology, Affiliated Hospital of Nantong University, Nantong, 226001 Jiangsu Province China; 4 Department of Neurology, Affiliated Hospital of Nantong University, Nantong, 226001 Jiangsu Province China

**Keywords:** Pannexin 1, Schwann cell, IL-6, Neuropathic pain, Inflammatory factor

## Abstract

**Background:**

Neuropathic pain is still a challenge for clinical treatment as a result of the comprehensive pathogenesis. Although emerging evidence demonstrates the pivotal role of glial cells in regulating neuropathic pain, the role of Schwann cells and their underlying mechanisms still need to be uncovered. Pannexin 1 (Panx 1), an important membrane channel for the release of ATP and inflammatory cytokines, as well as its activation in central glial cells, contributes to pain development. Here, we hypothesized that Schwann cell Panx 1 participates in the regulation of neuroinflammation and contributes to neuropathic pain.

**Methods:**

A mouse model of chronic constriction injury (CCI) in CD1 adult mice or P0-Cre transgenic mice, and in vitro cultured Schwann cells were used. Intrasciatic injection with Panx 1 blockers or the desired virus was used to knock down the expression of Panx 1. Mechanical and thermal sensitivity was assessed using Von Frey and a hot plate assay. The expression of Panx 1 was measured using qPCR, western blotting, and immunofluorescence. The production of cytokines was monitored through qPCR and enzyme-linked immunosorbent assay (ELISA). Panx1 channel activity was detected by ethidium bromide (EB) uptake.

**Results:**

CCI induced persistent neuroinflammatory responses and upregulation of Panx 1 in Schwann cells. Intrasciatic injection of Panx 1 blockers, carbenoxolone (CBX), probenecid, and Panx 1 mimetic peptide (^10^Panx) effectively reduced mechanical and heat hyperalgesia. Probenecid treatment of CCI-induced mice significantly reduced Panx 1 expression in Schwann cells, but not in dorsal root ganglion (DRG). In addition, Panx 1 knockdown in Schwann cells with Panx 1 shRNA-AAV in P0-Cre mice significantly reduced CCI-induced neuropathic pain. To determine whether Schwann cell Panx 1 participates in the regulation of neuroinflammation and contributes to neuropathic pain, we evaluated its effect in LPS-treated Schwann cells. We found that inhibition of Panx 1 via CBX and Panx 1-siRNA effectively attenuated the production of selective cytokines, as well as its mechanism of action being dependent on both Panx 1 channel activity and its expression.

**Conclusion:**

In this study, we found that CCI-related neuroinflammation correlates with Panx 1 activation in Schwann cells, indicating that inhibition of Panx 1 channels in Schwann cells reduces neuropathic pain through the suppression of neuroinflammatory responses.

**Supplementary Information:**

The online version contains supplementary material available at 10.1186/s12974-022-02603-x.

## Introduction

Neuropathic pain caused by nerve injury or related diseases is still a challenge for clinical treatment due to the complicated pathogenesis. Most of the underlying pathogenic mechanisms are focused on neuronal cells, including the excitability of primary sensory neurons and the imbalance between excitatory and inhibitory synaptic transmission within the CNS. However, accumulating evidence suggests that non-neuronal cells, especially glial cells, play a non-neglectful role in regulating neuropathic pain [1–3], most of which are studied in the brain, spinal cord, and primarily in astrocytes and microglia [4–6]. Schwann cells, the most prevalent glial cells in the PNS, act as physical support cells for axons, and release various signaling molecules that interact with the long axons [7]. After sciatic nerve injuries, first response cells Schwann cells will activate a series of changes, including migration, proliferation, and production of numerous factors that assist in repairing the injured axons [8, 9]. Furthermore, accumulating evidence suggests that multiple active molecules, receptors, and channels are released from Schwann cells [10–13]. Nevertheless, the underlying mechanisms of Schwan cells in the regulation of neuropathic pain remains little known.

Pannexins (Panx) consist of large-pore membrane channels and they are highly permeable to ATP and other signaling molecules. Among the three family  members (Panx 1, Panx 2, and Panx 3), Panx 1 is the most characterized and ubiquitously expressed within the nervous system as well as neurons and glial cells [14]. Moreover, Panx 1 activation in the dorsal root ganglion (DRG), astrocytes, and microglia participates in neuropathic pain development [15–17]. In addition, our previous study revealed that Panx 1 is highly expressed in Schwann cells compared with the other two family members and the inhibition of Panx 1 reduces hypotonicity-induced ATP release [18]. However, the contribution of Panx1 in Schwann cells regarding neuropathic pain is still unclear.

Knowledge of neuroinflammation in neuropathic pain has accumulated and yielded potential therapeutic targets [19–22]. Glial activation and cytokine production in neuroinflammation is believed to be vital mechanisms underlying the treatment of neuropathic pain [2, 13, 23–25]. This study found that chronic constriction injury (CCI) induces elevated expressions of inflammatory factors that persist for at least 2 weeks after the initial injury. Intrasciatic injection of pannexin 1 blockers, carbenoxolone (CBX), probenecid, and pannexin 1 mimetic peptide, ^10^Panx at 10 days after CCI, effectively reduced mechanical and heat hyperalgesia. Moreover, specific knockdown of Panx 1 with genetic manipulation significantly reduced CCI-induced neuropathic pain. To determine the neuroinflammatory mechanisms of Panx 1 in Schwann cells, LPS was used to mimic the conditions of neuroinflammation. The data revealed that LPS induces an increase in the production of pro-inflammatory factors that are inhibited by CBX and Panx 1 siRNA. In addition, LPS treatment had a minimal effect on Panx 1 expression; however, increased Panx 1 channel activity, which was significantly inhibited by Panx 1 blockers and Panx 1 siRNA. Together, our findings suggest that inhibition of Panx 1 in Schwann cells may be an effective approach in reducing neuropathic pain by down-regulating inflammatory responses.

## Methods

### Animals, surgery and virus injection

Adult male CD1 mice (6–8 weeks) and P0-Cre transgenic mice (6–8 weeks; Jackson Laboratory; stock no: 017927) were chosen for behavioral tests, and neonatal CD1 mice (1–2 days) were prepared for the collection of primary cultured Schwann cells. CCI, a model of neuropathic pain, was used in the study [26]. In brief, mice were anesthetized with isoflurane, and under a surgical microscope, three ligatures (1 mm distance between each ligature) were loosely tied around the left sciatic nerve (proximal to the trifurcation) until a short flick of the ipsilateral hind limb was observed. Control mice received the identical procedure but without nerve ligation. 10 days after CCI, intrasciatic injection of AAV2/9 vector into the nerve ligatures was performed with the syringe as previously described [9, 27]. The virus including AAV2/9-RNAi vector (pAAV-CBG-Dio-EGFP-miR30shRNA-WPRE; OBio Technology) with the desired shRNA or control sequence. Each mouse was injected unilaterally with 5 μl at a final titer of 2 × 10^10^ vg. Our study’s surgical preparations and experimental protocols were authorized by the Institution Animal Care & Use Committee of Nantong University.

### Behavioral test

To evaluate mechanical and thermal sensitivity, behavioral tests were performed as previously reported [28]. Before baseline tests were performed, animals were habituated to the test environment for 3 days. Behavioral tests of P0-Cre mice were carried out at 14 days after virus injection. To test mechanical sensitivity, mice were placed in boxes on an elevated metal mesh floor. After 60 min of habituation, a series of von Frey hairs (0.02–2.56 g; Stoelting) was used vertically to the plantar surface of each hind paw with logarithmically increasing stiffness and each hair test was lasted for 3–5 s. For thermal sensitivity, hot plate, and Hargreaves radiant heat apparatus (IITC Life Science) were applied following the manufacturer’s instructions. The baseline of paw withdrawal latency was set at 9–12 s, with 20 s used as the cut-off to avoid tissue damage. All behaviors were tested blindly.

### Primary Schwann cell cultures and transfection

Primary Schwann cell cultures were performed as previously described [18]. In brief, after isolation from the sciatic nerves, cells were incubated in DMEM containing 10% horse serum, and supplemented with 1 ng/ml heregulin β-1 (Peprotech, 100–03) and 0.5 μM forskolin (Sigma-Aldrich, F6886) for proliferation. Schwann cells were purified by digesting with 0.25% trypsin (Sigma-Aldrich) for short time (10–20 s) to obtain > 90% pure Schwann cells, as determined by immunocytochemistry with S100 β, a Schwann cell marker [29].

On the second day after plating, RNAiMAX (Invitrogen) was used to transfect the Schwann cells with small interfering RNA (siRNA), or negative control siRNA. Panx1 siRNA (GCCTCATTAACCTCATTGT) as well as the universal negative control (GAAGAGGUAUUGAAUGCUA) were purchased from RiboBio (Guangzhou, China).

### Drug and administration

All chemicals purchased from Sigma-Aldrich unless otherwise noted. Panx 1 mimetic peptide, ^10^Panx, and scrambled ^10^Panx were purchased from Tocris. Panx 1 inhibitors, carbenoxolone (CBX, 100 μM; Sigma, C4790), probenecid (500 μM; Sigma, P8761) were used to pretreat the cells for 60 min followed by 100 ng/ml LPS (Sigma, L2360) treatment for 24 h in cultured Schwann cells. For intrasciatic injection, CBX (100 μM, 6 μl), probenecid (500 μM, 6 μl), ^10^Panx (100 μM, 6 μl; Tocris, 3348) and scrambled ^10^Panx (100 μM, 6 μl; Tocris, 3708) were administrated into the mesoneurium of the left sciatic nerve with a 29-G ultra-fine needle at 10 days after CCI surgery. The negative controls were treated with vehicle solutions at the same volume.

### Quantitative real-time PCR (qPCR)

Sciatic nerves total RNA was isolated using a Trizol reagent (Invitrogen, 15596018), and cultured Schwann cells total RNA was extracted with an RNA quick purification kit (ESscience Biotech, RN001). The RNA was reversed transcribed using a Prime Script RT kit (TaKaRa, RR036A). The cDNA of the reverse transcribed RNA was used for the qPCR assay and the amplification curves were produced using SYBR Green PCR Master Mix (TaKaRa, RR420B) according to the manufacturer’s instructions. The relative levels of target mRNA were normalized to the 18S or GAPDH via the 2^−∆∆CT^ formula. Primers for qPCR in our study were synthesized by Thermo Fisher and listed in Table 1.

**Table 1:** .

Gene	Sense sequence	Anti-sense sequence
GAPDH	TCCATGACAACTTTGGCATTG	CAGTCTTCTGGGTGGCAGTGA
18S	GACAGGATTGACAGATTGATAG	CGTTATCGGAATTAACCAGAC
IL-1β	TGTCTTGGCCGAGGACTAAG	TGGGCTGGACTGTTTCTAATG
IL-6	TCCATCCAGTTGCCTTCTTGG	CCACGATTTCCCAGAGAACATG
IL-10	CTGGACAACATACTGCTAAC	AAATGCTCCTTGATTTCTGG
TNF-α	CCCCAAAGGGATGAGAAGTT	CACTTGGTGGTTTGCTACGA
TGF-β	CCTATTTAAGAACACCCACTTT	TCCTGAATAATTTGAGGTTGAG
Panx 1	CCTCATTAACCTCATTGTGTAT	CATTGTAGCCTTCAGACTTG
Panx 2	TGTGGTCTATACTCGCTATG	CTCCTGCTGGATGTCTAG
Panx 3	CTCAGATTATGGACTATGAACAC	TCAGAAGGTAACTTGGAGAAT
Caspase 3	TGGGCACATCTTCAGAAA	GTGGTAACTTGGACATCATC

### Immunostaining

Immunohistochemistry staining was conducted as previously described [9]. In brief, after anesthetizing the mice with isoflurane, the animals were perfused with saline followed by 4% PFA. The sciatic nerves and the DRG were removed and post-fixed in 4% PFA for 8–12 h. The sciatic nerve sections and DRG slices (12 μm) were sectioned using a cryostat machine followed by immunohistochemistry staining. After washing, the slices were permeabilized with 0.3% Triton X-100 for 10 min, and blocked with 10% BSA for 2 h at room temperature, and processed for immunostaining with the following primary antibodies: S100 β (1:400, mouse; Sigma, AMAB91038), NF200 (1:300, mouse; Sigma, N0142), Panx1 (1:200, rabbit; Abcam, ab124131) overnight at 4 °C. Then, the slices were incubated with a mixture of cy3 and 488-, or cy3 and cy5-conjugated secondary antibodies (1:1000; Jackson Immuno Research) for 2 h at room temperature. For the staining of neurons in the DRG slices, Nissl staining (1:200, Thermo, N-21483) was applied and performed via the manufacturer’s instructions. Stained samples were examined using a fluorescence microscope (Nikon), and images were captured with a CCD Spot camera.

### Western blotting

Tissues for western blotting analysis were collected from three ligatures of sciatic nerves at 4, 14 days post-CCI and that of the sham group. Cells for western blotting analysis were obtained from cultured Schwann cells with different treatments. Protein was extracted with a RIPA tissue/cell lysis buffer (Beyotime, P0013B) containing a cocktail of protease inhibitors (1 mg/ml, 1:00; Sigma, P8340). After total protein quantification by a BCA Protein Assay kit (Beyotime, P0010), a 10% SDS-PAGE was used to separate 20–30 μg total protein per well of each group as previously described [18]. Next, the protein gel was transferred to a PVDF membrane and blocked using 5% nonfat milk. The blots were then incubated with the following antibodies overnight at 4 °C: Panx 1 (1:200, rabbit; Abcam, ab124131) and GAPDH (1:10,000, mouse; Proteintech, 60004-1-Ig). The bolts were further incubated with horseradish peroxidase (HRP)-conjugated secondary antibody and developed in an ECL solution (Thermo Fisher, 32106). Chemiluminescence was conducted via a Bio-Rad ChemiDoc for 5–60 s. The densitometry of the selected bands was analyzed by NIH Image J software.

### Enzyme-like immunosorbent assay (ELISA)

Mouse IL-1β (FMS-ELM002) and IL-6 (FMS-ELM006) ELISA kits were provided by FCMACS. Protein samples from the treated Schwann cells were extracted and measured using the same methods used for western blotting. 20–30 μg proteins were applied to each reaction and performed as previously described [30]. A standard curve was conducted for each experiment.

### Ethidium bromide uptake

The procedure was performed as previously described [26]. After pretreatment with Panx 1 inhibitors or Panx 1-siRNA, Schwann cells were treated with 100 ng/ml LPS for 24 h followed by exposure to 0.5 μM ethidium bromide (Sigma, E1510) for 15 min and 30 min at 37 °C. After washing, cells were visualized by a Nikon fluorescence microscope, and images were captured on a CCD spot camera from three randomly chosen fields on each coverslip. The analysis of the integrated densities for ethidium bromide staining was performed using ImageJ software. The experiments were conducted in triplicate using different batches of cells.

### Cytokine assay

The mouse cytokine array kit (R&D Systems, ARY006) was utilized for the parallel determination of the selected mouse cytokines levels produced by the Schwann cells. After treatment, protein samples were prepared using the same method used for western blotting. 300 μg of protein from each reaction was collected from the different groups, including Ctrl, Ctrl-siRNA + LPS, and Panx1-siRNA + LPS groups. All procedures were conducted according to the manufacturer’s protocol. The images were captured and analyzed using the same methods described for western blotting.

### Statistical analysis

All results were presented as mean ± SEM from (*n*) experiments. Statistical comparisons between groups were assessed using Student's *t-*test or ANOVA, followed by Tukey’s multiple comparisons or Dunnett’s tests, and *p* < 0.05 was set as the statistical significance. Detailed statistical routes are shown in the figure legends.

## Results

### Chronic constriction injury induces persistent inflammatory responses

**Fig. 1:**
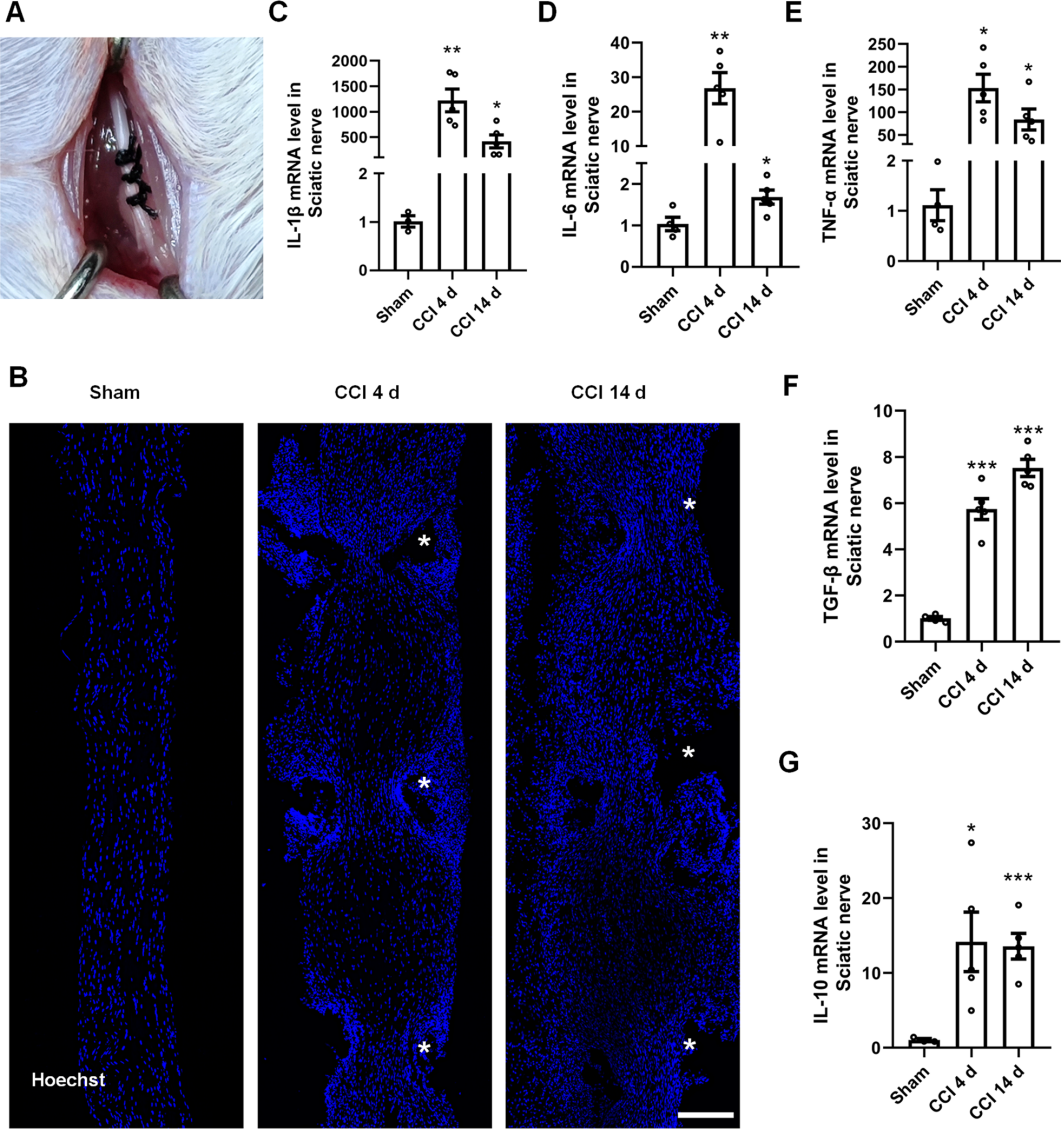
CCI induces persistent inflammatory responses. **A** Surgical image of the CCI model in mice. **B** Hoechst staining of the sciatic nerves at 4 and 14 days after CCI. White stars indicate the distribution of ligations. Scale bar = 100 μm. **C–G** Changes of inflammatory factors, including pro-inflammatory factors, IL-1β, IL-6 and TNF-α, and anti-inflammatory factor, TGF-β and IL-10 after CCI at 4 and 14 days. Data are mean ± SEM, *n* = 3–5 mice/group. *p < 0.05, **p < 0.01, ***p < 0.001 vs. the sham group. One-way ANOVA followed by Dunnett’s test

CCI is one of the most widely used models to study neuropathic pain [31]. CCI-induced neuropathic pain appears at 3–4 days and fully develops at 10 days post-surgery [32]. Neuroinflammation is a common reaction in CCI that contains glial activation as well as the production of neuroinflammatory factors [2, 20, 23]. Herein, we found that CCI (Fig. 1A) induces apparent inflammatory swelling between three ligations within the sciatic nerves at 4 and 14 days compared to the sham injury (Fig. 1B). We also performed a qPCR analysis to test the expression levels of CCI-induced inflammatory factors. The qPCR results showed that CCI significantly increases the expression of pro-inflammatory factors including IL-1β, IL-6 and TNF-α, and that of the anti-inflammatory factor IL-10 and TGF-β at 4 days. The inflammatory effects of CCI lasted for 14 days post-injury (Fig. 1C–G). The above data suggest that the CCI-induced inflammatory response is present at 4 days and persists for a minimum of 14 days.

### CCI increases the expression level of Panx 1 in Schwann cells

**Fig. 2:**
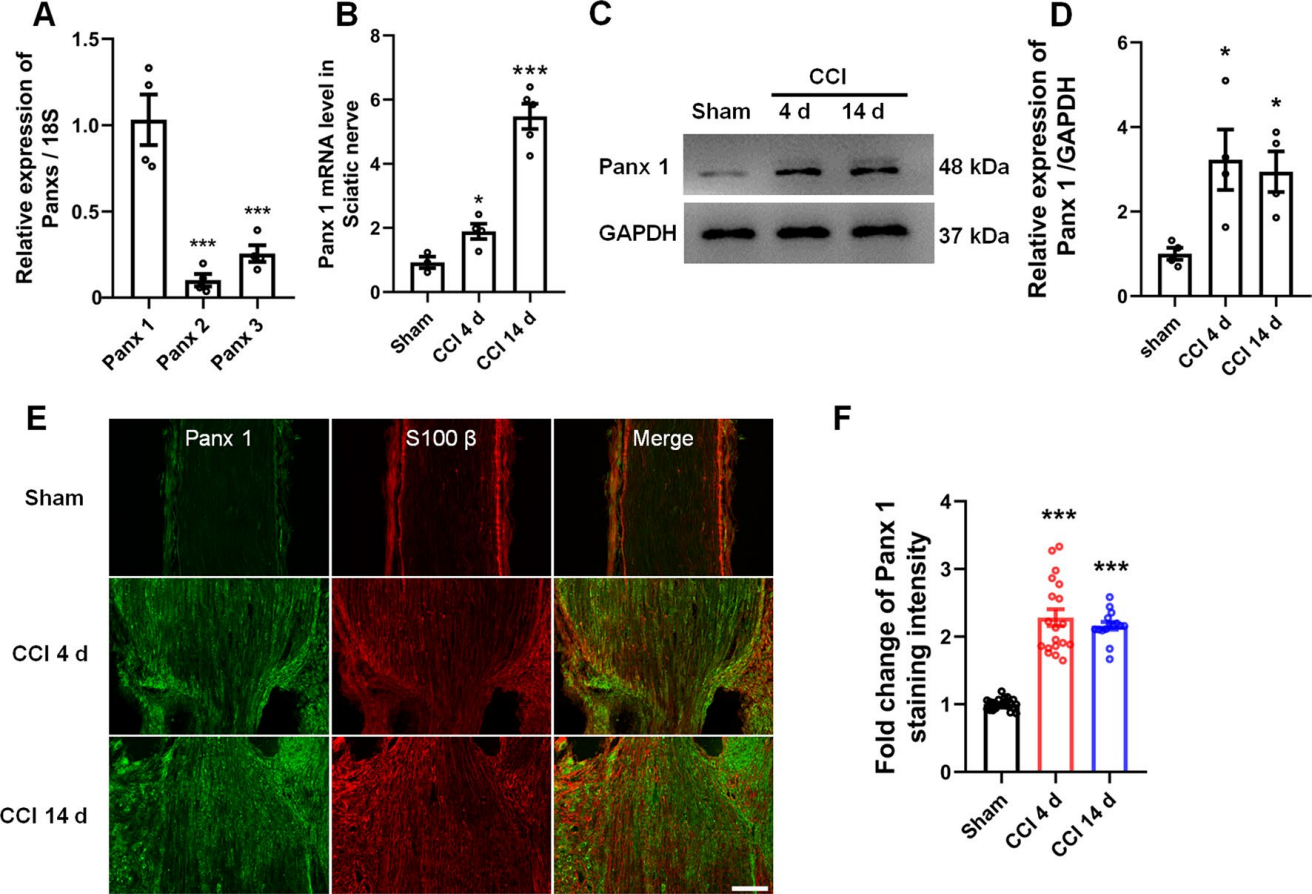
Nerve injury induces the upregulation of Panx 1 in Schwann cells. **A** The mRNA expression of Panx 1, Panx 2, and Panx 3 within the sciatic nerves of adult CD1 mice (6–8 weeks). **B–D** Panx 1 mRNA and protein were detected in sciatic nerves at 4 and 14 days post-CCI. **E** Double-immunofluorescence labeling with Panx 1 (green) and Schwann cells marker S100 β (red) after CCI at 4 and 14 days. Scale bar = 100 μm. **F** Quantification of Panx 1 immunostaining intensity in the sham group and the CCI groups. All data are mean ± SEM *n* = 4 mice/group. *p < 0.05, ***p < 0.001 vs. the sham group. One-way ANOVA followed by Dunnett’s test

According to our previous study, compared with Panx 1, Panx 2, and Panx 3 were less detectable within primary Schwann cell cultures [18]. We, therefore, examined the mRNA levels of different Panx proteins in sciatic nerves. The qPCR results showed that Panx 1 was the protein primarily expressed when compared with the other subtypes (Fig. 2A). After CCI, Panx 1 expression was significantly increased at both the mRNA and protein levels compared with sham surgery at 4 and 14 days (Fig. 2B–D). However, Panx 2 and Panx 3 mRNA had no significant increase after CCI at 4 and 14 days (Additional file 1: Fig. S1A-B). We next used a double immunostaining method to determine the distribution of Panx 1 within sciatic nerves. The results showed that Panx 1 had co-localized with the Schwann cell marker, S100 β (Fig. 2E), but not with the axonal marker, NF200 (Additional file 1: Fig. S2A). Moreover, the Nissl staining indicated that Panx 1 was primarily present around the soma of DRG neurons, but not in the axons (Additional file 2: Fig. S2B). Notably, quantification of immunohistochemistry staining in sciatic nerves confirmed that the CCI-induced Panx 1 expression levels are higher at 4 and 14 days post-CCI than that of the sham group (Fig. 2E, F). These results suggest that CCI-induction significantly up-regulates Panx 1 in sciatic nerves and is primarily distributed in Schwann cells.

### Inhibition of Panx 1 in Schwann cells reduces CCI-induced mechanical and thermal pain

**Fig. 3:**
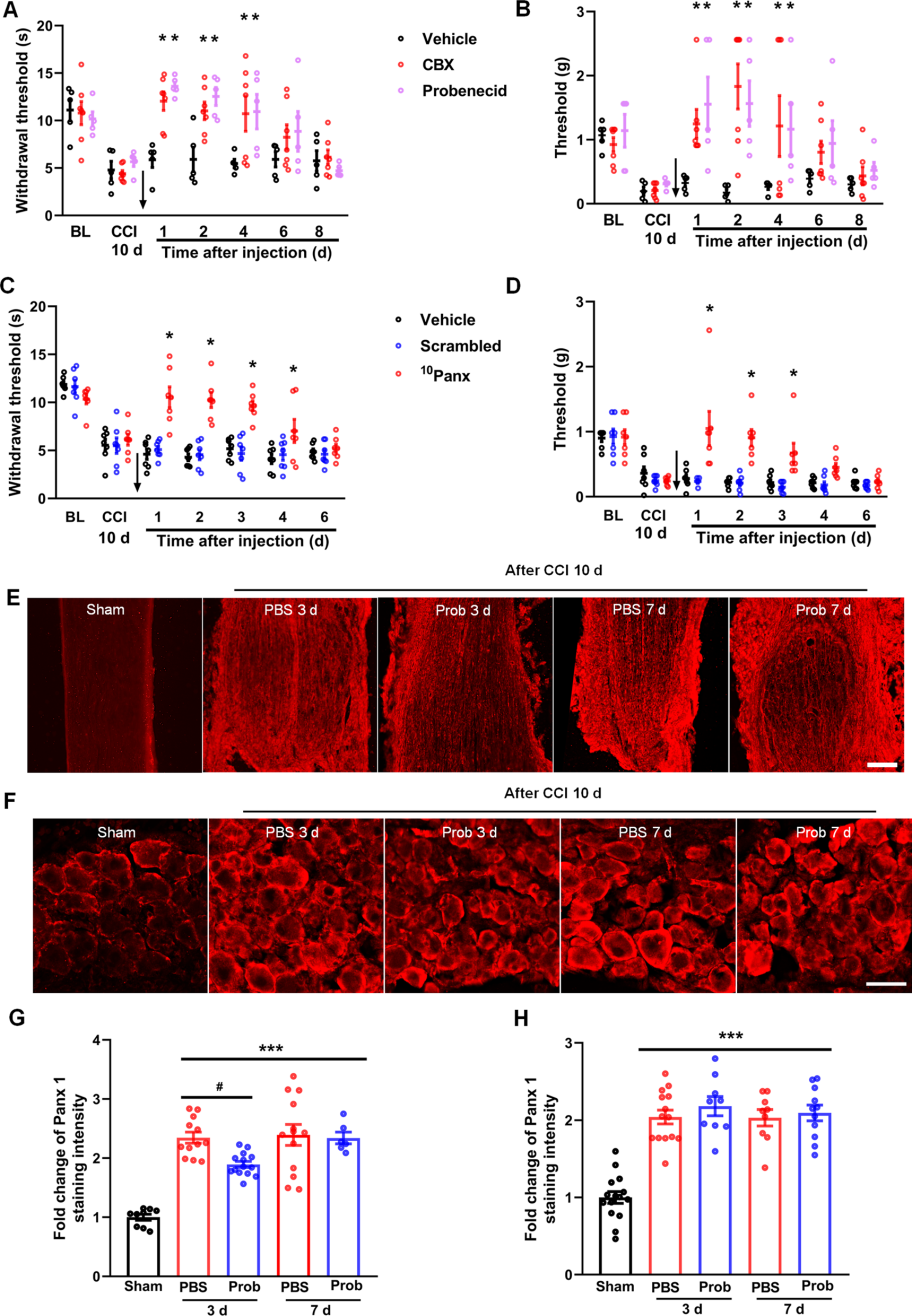
Injection of Panx 1 blockers reduces CCI-induced mechanical and thermal pain, and Panx 1 expression. **A, B** Intrasciatic injection of CBX (100 μM) and probenecid (100 μM) reverses CCI-induced mechanical and thermal pain *n* = 5 ~ 7 mice/group. **C, D** Sciatic nerve injection of Panx 1 mimetic peptide, ^10^Panx reduces mechanical pain for 3 days, and thermal pain for 4 days. However, the scrambled peptide had no effect. Arrows indicate the time-point of drug injection *n* = 7 mice/group. **E, F** Representative images of Panx 1 immunostaining at 3 and 7 days after injection with PBS or probenecid (Prob) after CCI in sciatic nerves (**E**) and the DRG (**F**). Scale bar = 100 μm. **G, H** Quantification of Panx 1 immunostaining intensity in different groups shown in **E** and **F**. *n* = 4 mice/group. All data are mean ± SEM. Two-way ANOVA followed by Tukey’s multiple comparison test (for behavioral results) or Dunnett’s test. *p < 0.05, vs. vehicle group, ***p < 0.001, vs. sham group and ^#^*p* < 0.05, vs. PBS group

Accordingly, CCI-induced neuropathic pain fully develops at 7–14 days [32]. In the present study, we therefore chose 10 days after CCI to examine mechanical and thermal pain. The results showed that the thresholds of neuropathic pain among the groups had no differences at 10 days post-CCI (Fig. 3A–D). We next used several pharmacological approaches to examine whether Panx 1 blockade can reverse neuropathic pain. First, we treated mice at 10 days after CCI with two pannexin inhibitors, CBX (100 μM, 6 μl) and probenecid (500 μM, 6 μl), and measured pain thresholds post-CCI days 1, 2, 4, 6, 8. As shown in Fig. 3A, B, pannexin inhibitors reversed mechanical and thermal allodynia at day 1 and persisted for at least 4 days after intrasciatic injection. Furthermore, intrasciatic injection of ^10^Panx, a Panx 1 mimetic peptide (100 μM, 6 μl) within the sciatic nerves 10 days after CCI significantly increases the mechanical threshold (lasting for 3 days) and the thermal threshold (lasting for 4 days) compared with the scrambled ^10^Panx (Fig. 3C, D).

Next, we selectively tested Panx 1 expression after treatment with the pannexin 1 inhibitor, probenecid. An approximate 20% decrease of Panx 1 expression in sciatic nerves was detected at 3 days after treatment with probenecid compared to treatment with PBS after CCI; however, no significant difference at day 7 after treatment was observed (Fig. 3E, G). To exclude any secondary effect of probenecid, the expression of Panx 1 in the DRG was detected after injection. The results showed that probenecid did not affect CCI-induced Panx 1 upregulation within the DRG (Fig. 3F, H). These findings suggest that intrasciatic injection of Panx 1 blockers contributes to the reduction of CCI-induced pain hypersensitivity as well as Panx 1 expression within the sciatic nerves.

**Fig. 4:**
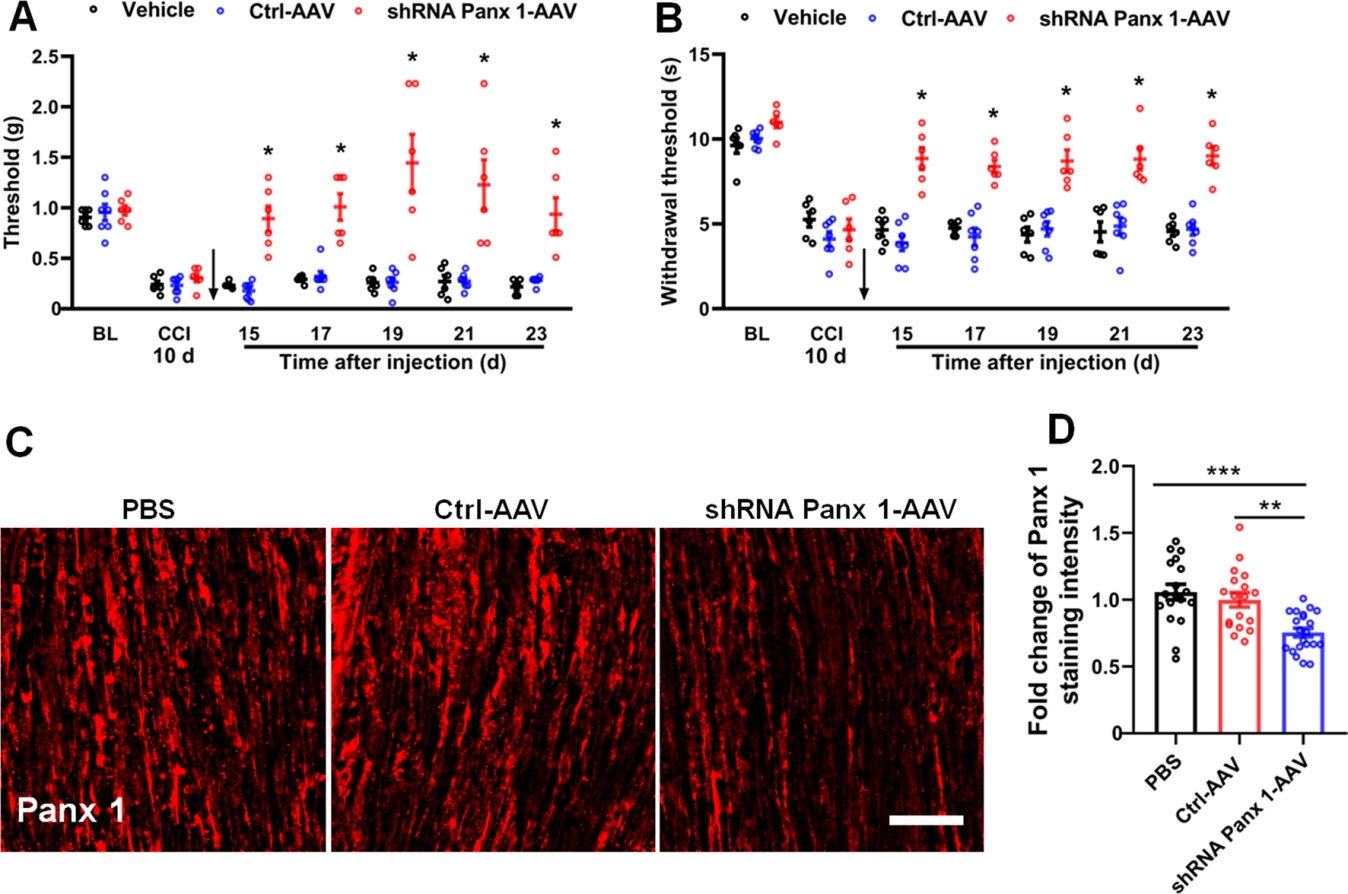
Specific knockdown of Panx 1 in Schwann cells reduces CCI-induced neuropathic pain. **A, B** Intrasciatic injection of shRNA Panx 1-AAV at 10 days post-CCI reduces CCI-induced mechanical and thermal pain, compared with vehicle and Ctrl-AAV groups. Arrows indicate the time-point of virus injection. *n* = 6–7 mice/group. **C**, **D** After behavioral tests (at 24 days post-CCI), the expression of Panx 1 was checked among three groups via immunofluorescent staining *n* = 3–4 mice/group. Scale bar = 50 μm. All data are mean ± SEM. Two-way ANOVA followed by Tukey’s multiple comparison test for behavioral tests. One-way ANOVA followed by Tukey’s multiple comparison test for immunostaining. *p < 0.05, vs. vehicle and Ctrl-AAV groups, **p < 0.001, vs. Ctrl-AAV group and ***p < 0.0001, vs. vehicle group

Literature has shown that Panx 1 is expressed in DRG, satellite cells, and macrophages within the peripheral nervous system [17, 33]. To further assess the role of Panx 1 in Schwann cells in CCI-induced neuropathic pain, we constructed an AAV-based Cre-dependent shRNA expression vector pAAV-CBG-Dio-EGFP-miR30shRNA (Panx 1)-WPRE (shRNA-Panx 1-AAV) or negative control AAV (Ctrl-AAV) and injected them into P0-Cre transgenic mice after 10 days of CCI. After 14 days, behavioral tests were carried out post-infection days 15, 17, 19, 21 and 23. The results showed that mechanical and thermal pain were significantly alleviated by the treatment with shRNA-Panx 1-AAV compared with the PBS and Ctrl-AAV groups, but no significant differences was detected between the PBS and Ctrl-AAV groups (Fig. 4A, B). We next killed the mice and checked the effect of AAV-infected cells with immunofluorescence staining, and the results showed that EGFP-labeled cells had a sound co-localization with S100 β-positive Schwann cells (Additional file 3: Fig. S3), and the expression of Panx 1 was downregulated in the group of shRNA-Panx 1-AAV, but not in the group of Ctrl-AAV (Fig. 4C, D). Therefore, these results indicate that specific knockdown of Panx 1 in Schwann cells reversed CCI-induced neuropathic pain.

### LPS induces the expression and release of inflammatory factors in Schwann cells through Panx 1

It has been shown that nerve injury can induce a lasting inflammatory response; thus, we explored whether Panx 1 was responsible for inflammatory responses in cultured primary Schwann cells. We used LPS to mimic an inflammatory stimulus to treat cultured Schwann cells. LPS-treated Schwann cells (0, 100, 500, and 1000 ng/ml) were treated for 24 h at 37˚C. The elevated IL-1β, IL-6, and TNF-α mRNA were observed in a dose-dependent manner; however, they did not affect the expression of TGF-β compared with baseline levels (Additional file 4: Fig. S4A-D). Furthermore, both an apoptotic marker, caspase 3 mRNA levels, and the cytomorphology of the Schwann cells showed no significant change after treatment with LPS at different doses (Additional file 4: Fig. S4E-F). Thus, we selected 100 ng/ml LPS as the stimulus to perform subsequent studies.

**Fig. 5:**
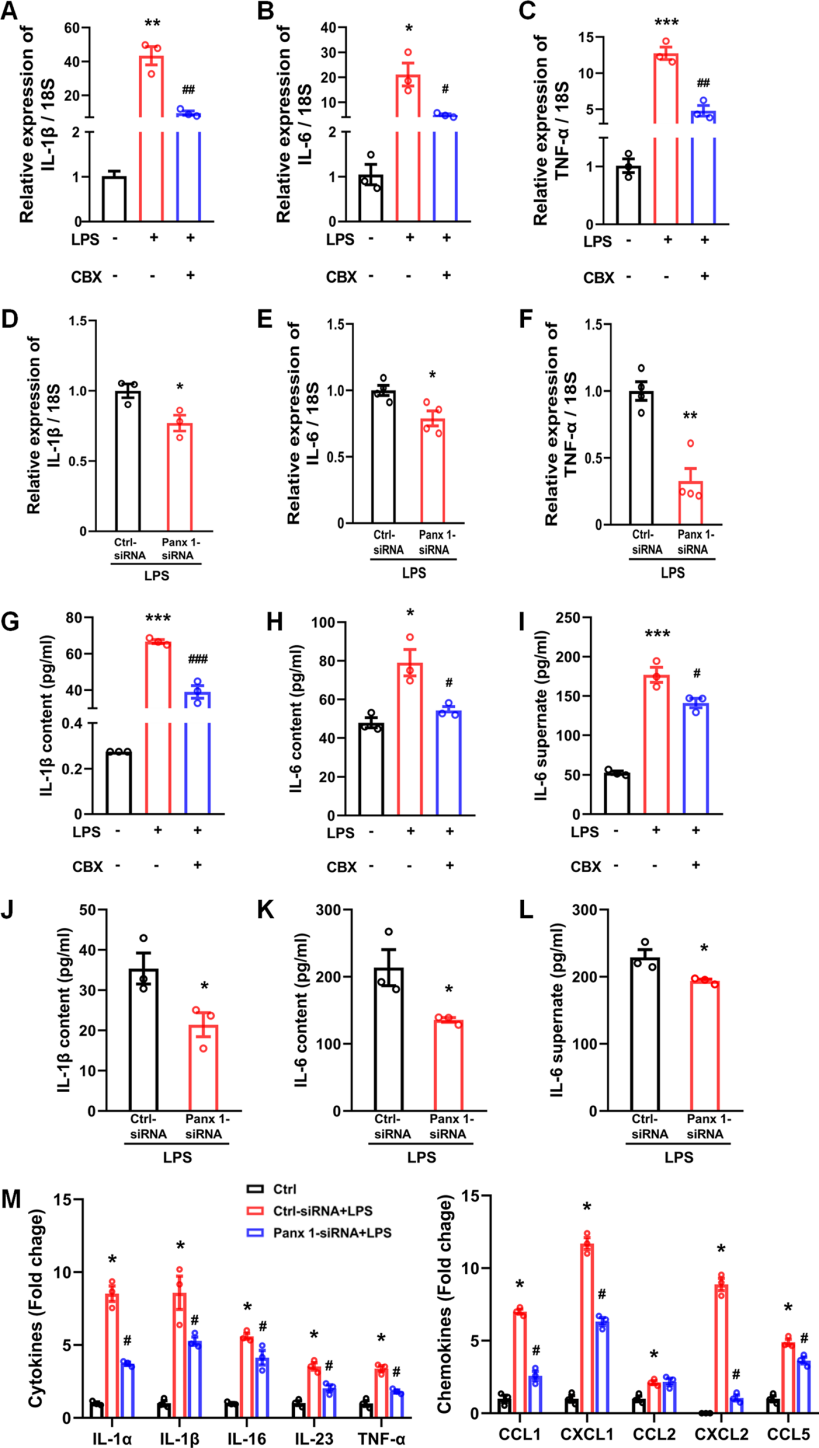
Both CBX and Panx 1 siRNA treatment reduces the production of inflammatory factors in LPS-treated Schwann cells. **A–C** Pretreatment with CBX (100 μM) attenuated LPS-induced increases in IL-1β, IL-6, TNF-α mRNA. *p < 0.05, **p < 0.01, ***p < 0.001, vs. control group, ^#^*p* < 0.05, ^##^*p* < 0.01, vs. the LPS treatment alone group *n* = 3. **D–F** LPS-induced increases of IL-1β, IL-6, and TNF-α mRNA was inhibited by Panx 1- siRNA, but not in the Ctrl-siRNA group. *p < 0.05, **p < 0.01, vs. Ctrl-siRNA group *n* = 3–4. **G, H, J, K** Both CBX and Panx 1-siRNA treatment reduced LPS-induced protein expression of IL-1β and IL-6. *p < 0.05, ***p < 0.001, vs. control or the Ctrl-siRNA group, ^#^*p* < 0.05, ^###^*p* < 0.001, vs. LPS treatment alone group. *n* = 3. **I**, **L** Reduced IL-6 release was observed in the groups pretreated with CBX or Panx 1 siRNA, but not in the Ctrl-siRNA group. *p < 0.05, ***p < 0.001, vs. control or Ctrl-siRNA group, ^#^*p* < 0.05, vs. LPS treatment alone group. *n* = 3. **M** Fold change of the expression of the selected cytokines and chemokines in different groups. *p < 0.05, vs. control, ^#^*p* < 0.05 vs. LPS + Ctrl-siRNA treatment group *n* = 3. The significance between the groups was analyzed using Student’s *t*-test (for two groups), or one-way ANOVA followed by Tukey’s multiple comparison test (for ELISA results) or Dunnett’s test

We next pretreated cultured Schwann cells with 100 μM CBX for 1 h. A qPCR assay was performed to assess the mRNA levels of various inflammatory factors. The results indicated that LPS-inducted cells increased IL-1β, IL-6, and TNF-α expression; however, CBX pretreatment significantly attenuated these levels (Fig. 5A–C). We further confirmed the effect of Panx 1 knockdown on cytokine expression in LPS-treated Schwann cells. The qPCR and western blotting assay results indicated that selective Panx 1 siRNA significantly attenuated Panx 1 expression under the control and LPS-treated Schwann cells conditions (Additional file 5: Fig. S5A-C, F), but did not affect Panx 2 and Panx 3 mRNA levels (Additional file 5: Fig. S5D-E). Furthermore, 100 ng/ml LPS-induced the increase of IL-1β, IL-6, and TNF-α expression was significantly inhibited by Panx 1 siRNA silencing (Fig. 5D–F). In addition, there was a minimal effect on the expression of TGF-β when treated with CBX or siRNA in LPS-treated Schwann cells (Additional file 6: Fig. S6A-B). Consistently, the CBX or Panx 1 siRNA also inhibited the cytosolic content of IL-1β, and IL-6, indicating the decreased expression of these cytokines at a protein level (Fig. 5G, H, J, K). Interestingly, inhibition of Panx 1 also attenuated IL-6 release induced by LPS treatment in Schwann cells (Fig. 5I, L), but we did not observe the release of IL-1β from Schwann cells with LPS treatment alone (data not shown).

To further demonstrate the effect of Panx 1 on cytokines expression, a mouse cytokine array containing 40 different cytokines/chemokines was utilized in LPS-treated Schwann cells. When comparing the cytokine levels in the protein samples from different treatments, we observed a significant increase in the expression of 5 inflammatory factors (IL-1α, IL-1β, TNF-α, IL-16, and IL-23,) and 5 chemokines (CCL1, CCL2, CCL5, CXCL1, and CXCL2) after treatment with LPS compared with the baseline levels (Additional file 7: Fig. S7A-B, and 5M). However, pretreatment of cultured Schwann cells with Panx 1 siRNA significantly suppresses the LPS-induced expression of the above factors (Fig. 5M). Of note, the expression levels of the positive controls did not change after treatment (Additional file 7: Fig. S7B). Taken together, these results suggest that Schwann cells Panx 1 is involved in the LPS-treated inflammatory responses.

### LPS increases the activity of Panx 1 but not of the expression

**Fig. 6:**
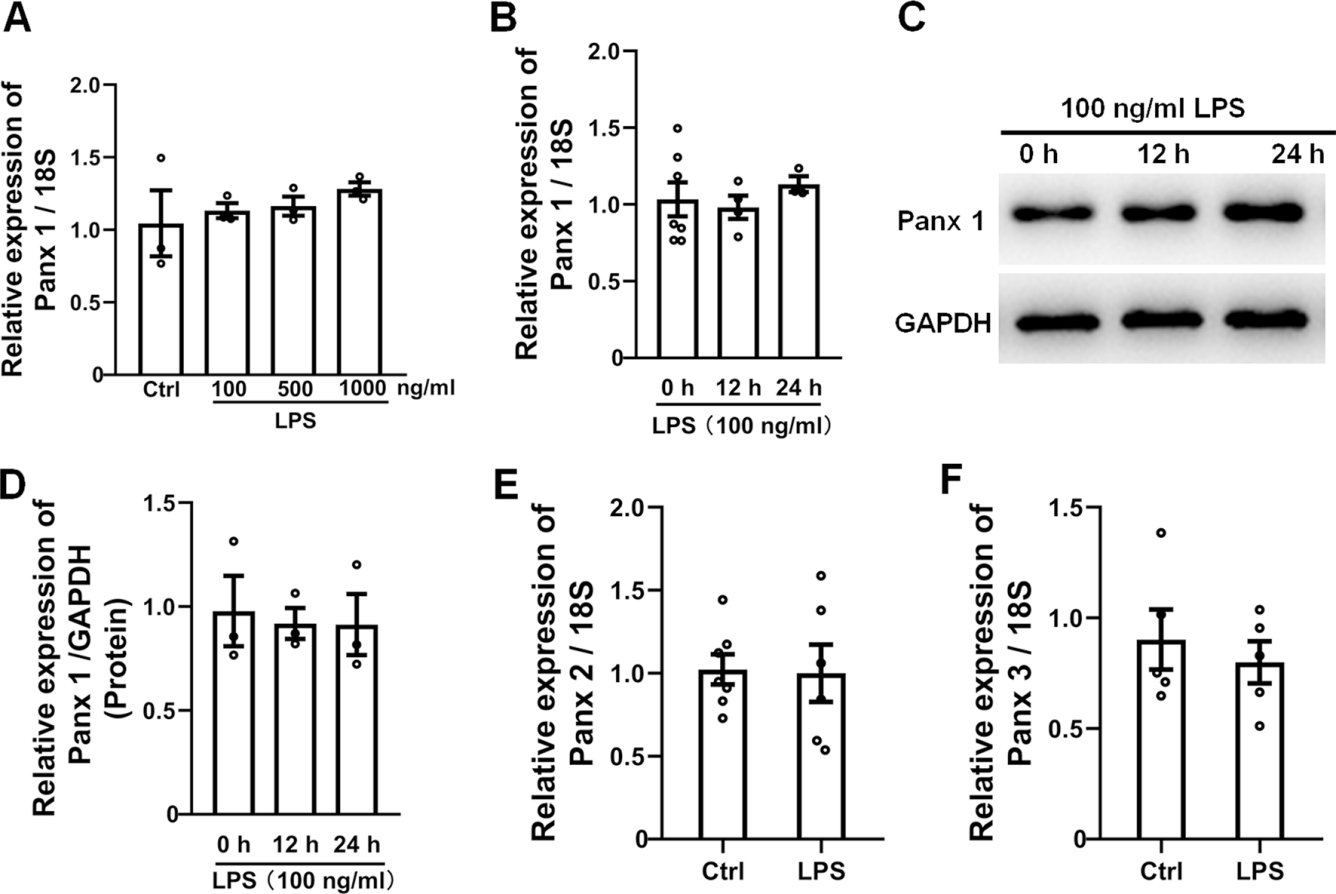
LPS does not affect Panx 1 expression in cultured Schwann cells. **A** Panx 1 mRNA expression levels was determined by qPCR in LPS-treated Schwann cells (0, 100, 500, and 1000 ng/ml, 24 h at 37 °C). **B–D** Determination of Panx 1 mRNA and protein levels at 0 h, 12 h, and 24 h after LPS (100 ng/ml) treatment in Schwann cells. **E, F** The determination of Panx 2 and Panx 3 mRNA levels via qPCR at 24 h in post-LPS (100 ng/ml) treated Schwann cells. Each experiment was performed in triplicate. Data are presented as mean ± SEM

Panx 1 mRNA and protein were elevated after CCI-induced nerve injury. To determine how Panx 1 regulates the LPS-treated inflammatory responses in Schwan cells, we performed qPCR and western blotting assays for Panx 1 expression. Our results showed that LPS treatment at different doses (0, 100, 500, and 1000 ng/ml) did not affect Panx 1 mRNA compared with the baseline levels (Fig. 6A). Moreover, no change was observed in Panx 1 mRNA and protein levels at 12 h and 24 h post-treatment with 100 ng/ml LPS compared with 0 h (Fig. 6B–D). We also found a minimal effect on Panx 2 and Panx 3 mRNA in the LPS-treated Schwann cells (Fig. 6E, F).

**Fig. 7:**
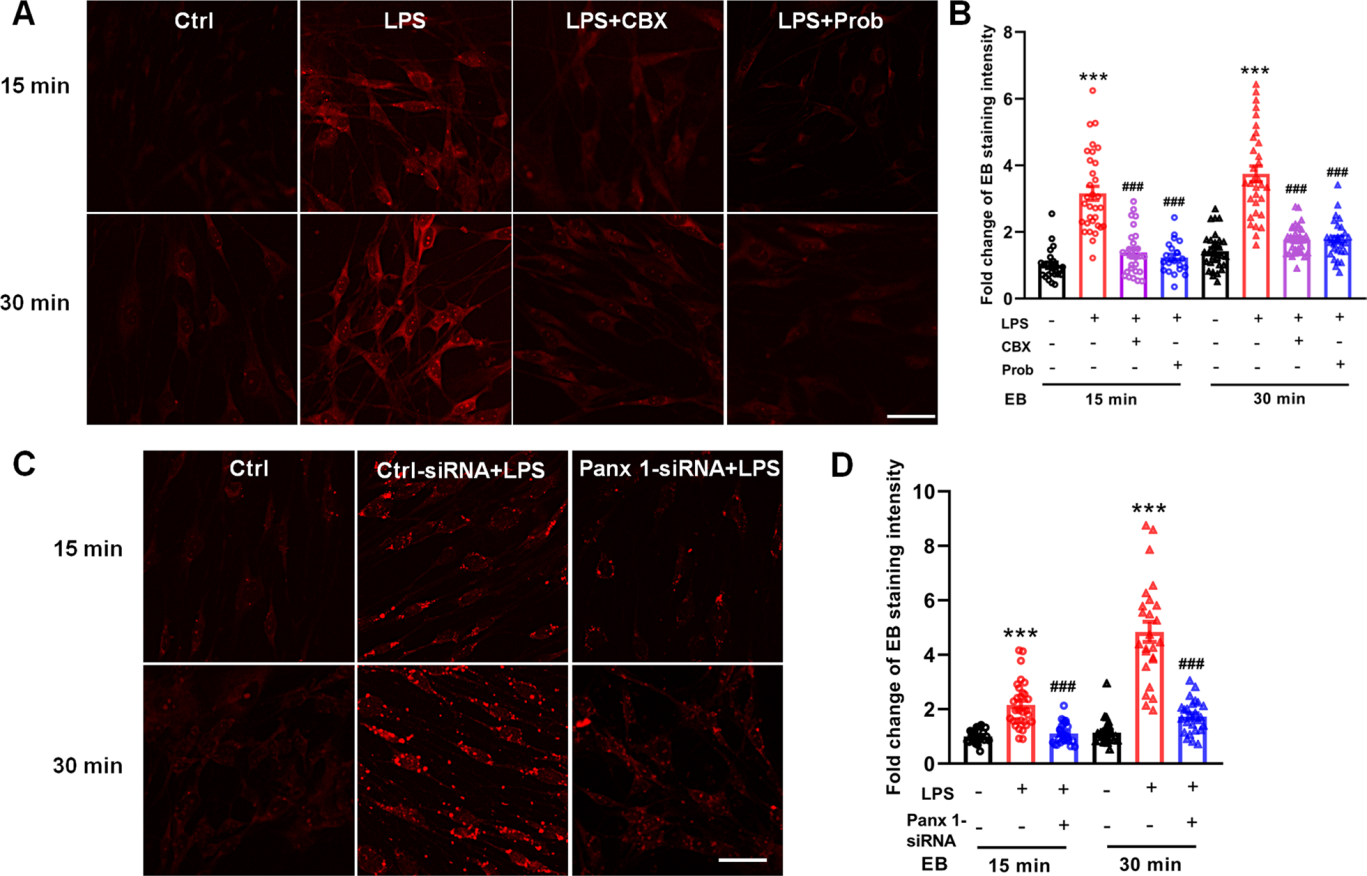
LPS-induced ethidium bromide uptake is dependent on the activity and expression of Panx 1 in cultured Schwann cells. **A, C** Ethidium bromide fluorescence in Schwann cells increased after exposure to LPS (24 h, 100 ng/ml); however, this increase was suppressed by the Panx 1 inhibitors, CBX and probenecid **(A)**, and Panx 1 siRNA **(C)**. **B, D** Quantification of ethidium bromide fluorescence intensities and the effect of Panx 1 inhibitors **(B)** and siRNA **(D)**. All data are mean ± SEM. ***p < 0.01, vs. the control group or Ctrl-siRNA group, ^###^*p* < 0.001, vs. LPS treatment alone group. The data were analyzed by one-way ANOVA followed by Tukey’s multiple comparison test

Panx 1, is a hemichannel, and its activity plays a vital role in allowing the cytoplasmic exchange with the extracellular space [34, 35]. Ethidium bromide uptake assay was used to index Panx 1 activity in glial cells [26, 35]. Therefore, we adopted this approach to test the activity of Panx 1 in LPS-treatment conditions. We added ethidium bromide into the medium of LPS-treated Schwann cells (100 ng/ml, 24 h), and found that a significant increase in the integrated densities of ethidium fluorescence appeared at both 15 min and 30 min compared with the control group (Fig. 7A–D). Of note, pretreatment with Panx 1 inhibitors, CBX, probenecid, and Panx 1 siRNA significantly suppressed the LPS-induced uptake of ethidium bromide, but not in the group of Ctrl-siRNA (Fig. 7A–D). These findings suggest that LPS treatment increases Panx 1 activity but not Panx 1 expression.

## Discussion

Our study has demonstrated the role of Schwann cells Panx 1 in a CCI-induced neuropathic pain model through the regulation of neuroinflammation. First, CCI induced a persistent (> 14 days) inflammatory process and the upregulation of Panx 1 within sciatic nerves after CCI surgery. Panx 1 is primarily distributed in Schwann cells but not in the axons of the sciatic nerves. Second, intrasciatic injection of CBX, probenecid, as well as the blocking peptide ^10^Panx effectively reduced CCI-induced mechanical and heat hyperalgesia. Moreover, specific knockdown of Panx 1 in Schwann cells with genetic manipulation significantly reduced CCI-induced neuropathic pain. Third, inhibition of Panx 1 with CBX and siRNA-Panx 1 was sufficient to suppress both the expression and release of inflammatory factors in LPS-treated Schwann cells. Finally, LPS induced an increase in Panx 1 channel activity but did not affect Panx 1 expression.

Neuroinflammation and glial activation caused by nerve injury is associated with Panx 1 activation [19, 22, 24]. Furthermore, upregulation of inflammatory factors within sciatic nerves was observed at 4 days and lasted for 14 days after CCI. Our previous study revealed that higher Panx 1 mRNA levels in cultured Schwann cells were detected compared with Panx 2 and Panx 3 [18]. Herein, we characterized pannexins expression in sciatic nerves, and confirmed that the level of Panx 1 is higher than the other subtypes and CCI increases Panx 1 mRNA and protein levels. In contrast, lower expression of Panx 2 and Panx 3 was present after nerve injury. In addition, Panx 1 was primarily co-localized with the Schwann cell marker s100 β but not with the axonal marker, NF200 [17]. Therefore, the change of Panx 1 expression after nerve injury primarily occurs in Schwann cells indicating its potential role in CCI-induced nerve injury.

Due to Panx 1 broad expression within neurons, glial cells, and immune cells, it has been reported as an essential player in various types of pain, including neuropathic pain [16, 17, 36–40]. With intrathecal administration of Panx 1 blockers, CBX, and probenecid, it was first verified that inhibition of Panx 1 in sural nerve-transected rats significantly reduced mechanical hyperalgesia and wind-up activity [36]. In a recent study using targeted deletion of Panx 1 from neurofilament-H positive neurons and GFAP-positive glial cells, the researchers were the first to examine Panx 1 contribution in Panx 1-related transgenic mice. These findings reveal that Panx 1 contributes to the development of allodynia in orofacial pain and that glial and neuronal Panx 1 has different effects on the baseline threshold and the duration of tactile hypersensitivity [41]. In neuropathic pain from CCI and spared nerve injury, hypersensitivity was profoundly attenuated by Panx 1 deletion; however, this hypersensitivity was restored after receiving bone marrow-derived cells expressing Panx 1 [39]. In addition, inhibition of Panx 1 upregulation in the DRG after spinal nerve ligation significantly reduced pain hypersensitivity through intrathecal injection of Panx 1-specific siRNA or Panx 1 blockers [17]. Although emerging evidence suggests that inhibition of Panx 1 from glial, neuronal, and immune cells is potent in relieving neuropathic pain, the role of Schwann cells, as first response cells for nerve injury remains unclear. We found that inhibition of Panx 1 with CBX or probenecid had a potential effect on alleviating neuropathic pain, and probenecid, an anti-gout medication reduced CCI-induced Panx 1 upregulation; however, did not affect Panx 1 expression in the DRG [42]. Moreover, specific knockdown of Panx 1 in Schwann cells by genetic manipulation attenuated CCI-induced neuropathic pain. We thus hypothesize that inhibition of Panx 1 in Schwann cells may contribute to the amelioration of neuropathic pain.

Activation of Panx 1 during the onset and development of disease caused the algogenic release of ATP as well as cytokine production. In spinal cord astrocytes, fibroblast growth factor-1 triggered Panx 1 opening and releasing pro-inflammatory factors to activate microglia, and further enhance the inflammatory responses through P2X7 receptor activation [43]. In microglia, blocking Panx 1 activity had a negative effect on ATP release and alleviated the severity of morphine withdrawal [37]. Moreover, inhibition of microglial Panx 1 reduced chronic joint pain by abrogating the release of the pro-inflammatory cytokine IL-1β [16]. As the principal glial cells of the PNS, Schwann cells were activated by nerve injury. In addition to proliferation and migration, they also produced various pain-related signaling molecules [13]. Our previous studies have shown that Panx 1 channels in Schwann cells mediate ATP release during hypotonicity stimulus [18]. Therefore, we hypothesize that Panx 1 in Schwann cells may be a target regulating the production and release of inflammatory cytokines during neuroinflammation.

LPS is a prototypical ligand of toll-like receptors (TLRs) and is widely used to activate glial cells [44, 45]. Schwann cells highly express subtypes of TLRs, including TLR 2, TLR3, and TLR4 [46]. We found that LPS treatment had a dose-dependent effect on the expression of pro-inflammatory factors. Of note, prenatal LPS exposure activated Panx 1 opening in offspring astrocytes to release IL-1β, TNF-α, as well as microglial-dependent production of inflammatory factors [47, 48]. Our study showed that inhibition of Panx1 either through CBX or Panx1-siRNA had a negative effect on the production of IL-1β, IL-6, and TNF-α, but not TGF-β. Moreover, cytokine expression of CBX-treatment alone in cultured astrocytes had no significant difference with baseline levels [30]. We selectively detected protein synthesis and secretion of IL-1β, and IL-6. The results showed a decrease in the concentrations of IL-6 both in the cytosolic content and in the supernatant after treatment with CBX or Panx1-siRNA within LPS-treated Schwann cells. Interestingly, only the cytosolic content of IL-1β was blocked after inhibition of Panx 1, and the release of IL-1β was not detected in LPS-treated Schwann cells. LPS induced the synthesis of the inactive and precursor forms (pro-IL-1β) of cytosolic IL-1β. The precursor form of IL-1β needs a secretory stimulus to become active, such as ATP to promote the synthesis of its active form and its cellular release [49, 50]. Therefore, compared with IL-1β release, the release of IL-6 is more dependent on the role of Panx 1 in LPS-treated Schwann cells. IL-6, a pro-inflammatory factor is involved in the development of pathological pain associated with inflammation, cancer, and nerve trauma [51–53]. Elevated expression of IL-6, IL-6R (gp80), and its signal transducing membrane protein gp130 were observed in experimental pain models. Moreover, the administration of IL-6-induced pain-related behaviors is attenuated in IL-6 knock-out mice and under the administration of anti-IL-6R antibodies [53, 54]. These findings, together with our study, suggest that IL-6 can be considered as a potential candidate in the management of neuropathic pain.

According to the cytokine array results in LPS-treated Schwann cells, besides IL-1β and TNF-α, the other cytokines and chemokines were observed and could act as players in various kinds of pain models. Up-regulation of IL-16 after sciatic nerve injury induces nociceptive hypersensitivity via kappa B beta dependent signaling [55]. IL-23 within rat spinal cords contributes to persistent allodynia through CX3CL1 and IL-18 signaling interactions after tetanic sciatic stimulation [56]. Glial cell-derived chemokine CCL1 and CXCL1 act upon the receptors CCR and CXCR2 in spinal neurons in order to increase neuronal excitability and modulate mechanical hypersensitivity [57–59]. In addition, the absence of CCL5 significantly attenuates partial sciatic nerve ligation-induced behavior hypersensitivity [60]. Moreover, these cytokines and chemokines contribute to the recruitment of monocytes and macrophages to sites of neuroinflammation [57, 60]. It is worth mentioning that inhibition of Schwann cell-derived CXCL1 leads to a decrease in human immunodeficiency virus type1 gp120-induced neuropathic pain as well as macrophage infiltration [61].

Nerve injury induces the upregulation of Panx 1 mRNA and protein levels; however, LPS treatment did not affect Panx 1 expression in cultured Schwann cells. These results indicate that Panx 1 induction in Schwann cells may depend on a much more complex mechanism; however, stimulation with LPS in vitro could not recapitulate the conditions of nerve injury. In addition, LPS effectively enhances the expression of Panx 1 in macrophages and monocytes, indicating the divergent effects of the cellular source of Panx 1 during treatment or active disease states [33]. However, downregulation of Panx 1 with siRNA or inhibition of its activity with CBX or probenecid profoundly inhibited LPS-induced ethidium bromide uptake, the results of which are consistent in astrocytes and microglia [47, 62]. These findings suggest that both the expression and activity of Panx 1 are important in response to LPS within Schwann cells.

In summary, we demonstrate the role of Panx 1 activity in relation to neuroinflammation and neuropathic pain via in vivo and in vitro experiments; however, our previous study found that compared with other P2X receptors, numerous P2X7 receptors were observed in Schwann cells [9]. Moreover, emerging evidence indicates that the interaction of Panx 1 with the P2X7 receptor plays a vital role in pain sensing both in the peripheral and the central nervous systems [63–67]. Therefore, the P2X7 receptor may be a key candidate within Schwann cell signaling pathways that interplay with Panx 1 in neuropathic pain. In addition, despite the fact that toll-like receptors, and LPS sensor molecules are known to be highly expressed in Schwann cells [68], how LPS-treated Schwann cells release cytokines and chemokines and are associated with their receptors (such as IL-1/IL1R, IL-6/IL-6R, CCL2/CCR2, and CXCL1/CXCR2) of the surrounding cells (neurons, monocytes and macrophages), and their underlying molecular mechanisms of neuropathic pain merit future studies.

## Conclusions

This study illustrated that Panx 1 is primarily distributed in Schwann cells and inhibition of Panx 1 ameliorates CCI-induced neuropathic pain. In LPS-treated Schwann cells, Panx 1 participated in regulating the production of pro-inflammatory factors, which is dependent on both the channel activity and the expression levels of Panx 1. The in vivo and in vitro results combined implicate a therapeutic potential of Schwann cell Panx 1 against neuroinflammation-associated neuropathic pain.

## Supplementary Information

**Additional file 1: Figure S1. **Panx 2 and Panx 3 mRNA expression after CCI. A-B. A qPCR assay was used to detect Panx 2 (A) and Panx 3 (B) mRNA levels at 4 and 14 days post-CCI. The data are mean ± SEM. *n* = 3 mice/group. **p < 0.01, ***p < 0.001, vs. sham injury. The data were analyzed by one-way ANOVA followed by Dunnett’s test.

**Additional file 2: Figure S2. **The distribution of Panx 1 in the DRG and within sciatic nerves. A. Double-immunofluorescence images of Panx 1 (green) and the sciatic nerve axonal marker, NF200 (red). (B). Images of Panx 1 immunostaining (red) and, Nissl staining (green) in the DRG; Scale bar = 50 μm. Enlarged images in lower panel are from the inset boxes of upper panel. Upper panel, scale bar = 100 μm. Lower panel, scale bar = 50 μm.

**Additional file 3: Figure S3. **ShRNA Panx 1-AAV or Ctrl-AAV-labeled EGFP-positive cells have a sound co-localization with S100 β-labeled Schwann cells. Enlarged images in lower panel are from the inset boxes of upper panel. Upper panel, scale bar = 50 μm. Lower panel, scale bar = 25 μm. *n* = 3–4 mice/group.

**Additional file 4: Figure S4. **The responses of LPS-treated cultured Schwann cells. A-E. mRNA expression levels of the inflammatory factors, IL-1β, IL-6, TNF-α, and TGF-β (A-D), the apoptosis marker, caspase 3 (E) and in LPS-treated Schwann cells (0, 100, 500, and 1000 ng/ml, 24 h at 37℃). F. Cytomorphology of LPS-treated Schwann cells at different doses (0, 100, 500, and 1000 ng/ml) under a light microscope. All data are mean ± SEM. *n* = 3. The data were analyzed by one-way ANOVA followed by Tukey’s multiple comparison test. *p < 0.05, **p < 0.01, ***p < 0.001, vs. the control group. Scale bar = 100 μm.

**Additional file 5: Figure S5. **The effect of Panx 1-siRNA on the expression of Panx 1 in Schwann cells. A, D, and E. mRNA expression of Panx 1, Panx 2, and Panx 3 after treatment with Panx 1 siRNA. B-C. Gel images and quantification of Panx 1 protein levels after treatment with Panx 1 siRNA. F. Panx 1-specific siRNA decreased Panx 1 mRNA expression levels under LPS treatment in Schwann cells. All data are mean ± SEM. *n* = 3. Two groups in A and C were analyzed with Student’s *t*-test. The differences between the three groups in F were analyzed using one-way ANOVA following by Tukey’s multiple comparison test. *p < 0.05, **p < 0.01, vs. the control group. ***p < 0.001, vs. the LPS treatment alone group.

**Additional file 6: Figure S6. **CBX and Panx 1-siRNA do not affect TGF-β mRNA expression in LPS-treated Schwann cells. A-B. Pretreatment with CBX (100 mM) for 2 h or Panx 1 siRNA for 36 h, qPCR was used to determine TGF-β mRNA expression levels in LPS-treated Schwann cells. All data are mean ± SEM. *n* = 4.

**Additional file 7: Figure S7. **Cytokine array reveals Panx 1-dependent cytokines and chemokines in LPS-treated Schwann cells. A. Coordinates of the cytokine array containing 40 different cytokines and chemokines, including three positive control (PC) proteins. B. Array membranes of protein expression among Ctrl, Ctrl-siRNA, and Panx 1-siRNA groups in LPS-treated Schwann cells.

## Data Availability

Please contact the author for data requests.

## Abbreviations

Panx 1: Pannexin 1
CCI: Chronic constriction injury
CBX: Carbenoxolone
IL-1β: Interleukin-1 beta
IL-6: Interleukin-6
TNF-α: Tumor necrosis factor α
IL-10: Interleukin-10
TGF-β: Transforming growth factor-β
ELISA: Enzyme-linked immunosorbent assay
DRG: Dorsal root ganglion
EB: Ethidium bromide
