# Supplementary figures and images for: Inhibition of Schwann cell pannexin 1 attenuates neuropathic pain through the suppression of inflammatory responses
Source: J Neuroinflammation. 2022 Oct 4;19:244. doi: 10.1186/s12974-022-02603-x (PMC9531429; doi:10.1186/s12974-022-02603-x)

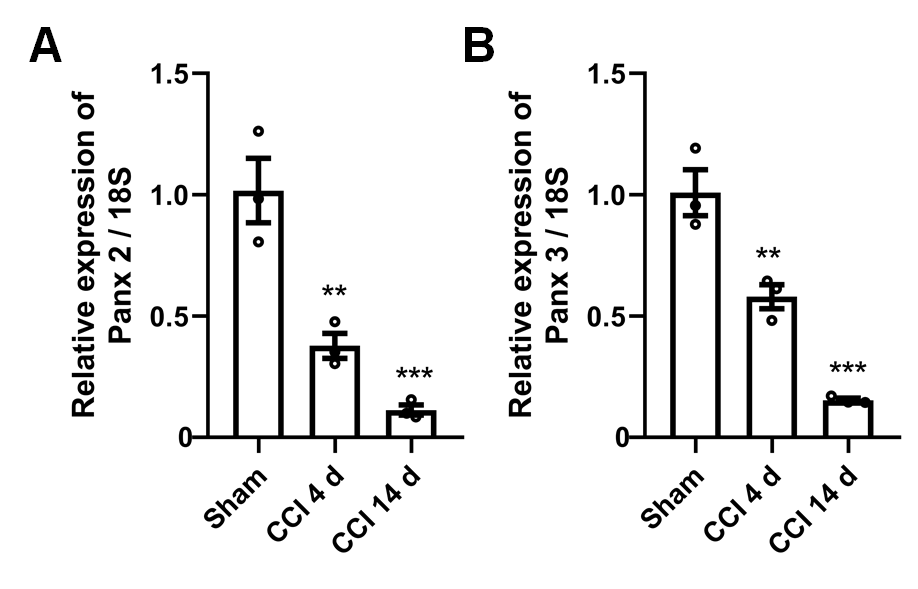

Supplement: Supplementary file 1 — Additional file 1: Figure S1. Panx 2 and Panx 3 mRNA expression after CCI. A-B. A qPCR assay was used to detect Panx 2 (A) and Panx 3 (B) mRNA levels at 4 and 14 days post-CCI. The data are mean ± SEM. n = 3 mice/group. **p < 0.01, ***p < 0.001, vs. sham injury. The data were analyzed by one-way ANOVA followed by Dunnett’s test. [file 12974_2022_2603_MOESM1_ESM.tif]

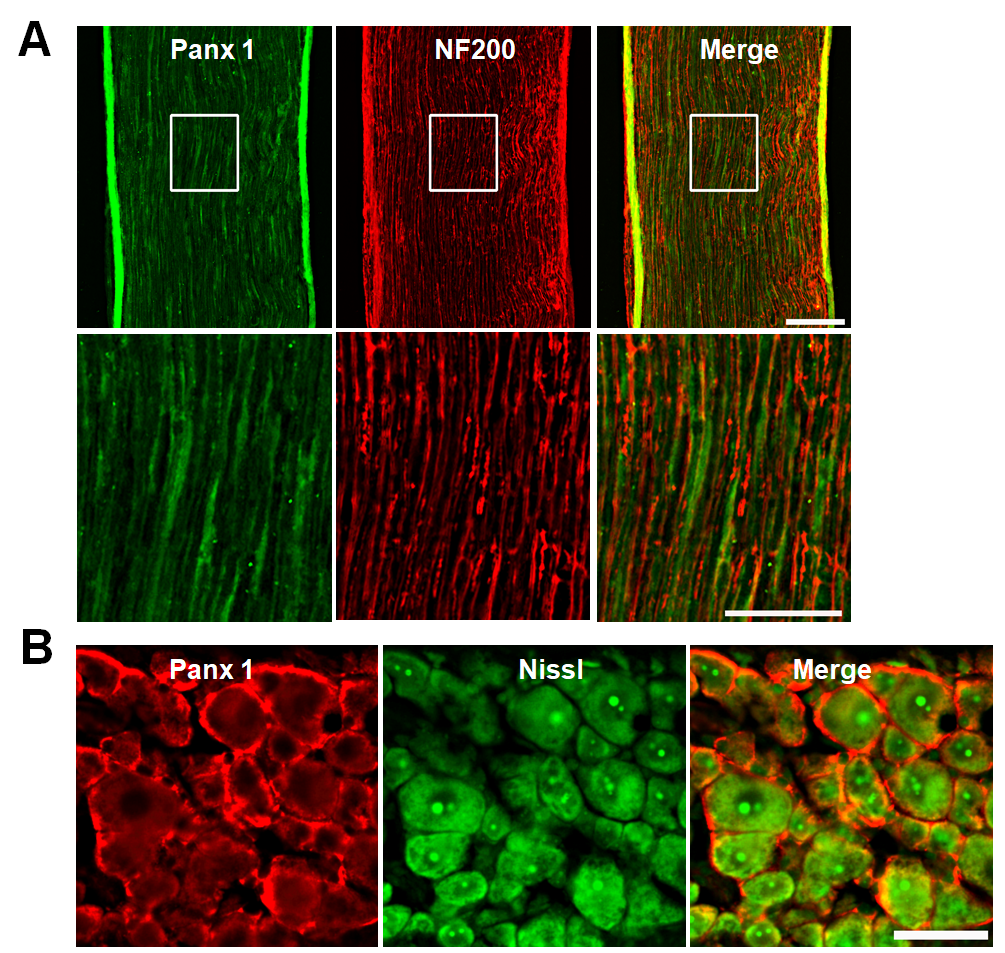

Supplement: Supplementary file 2 — Additional file 2: Figure S2. The distribution of Panx 1 in the DRG and within sciatic nerves. A. Double-immunofluorescence images of Panx 1 (green) and the sciatic nerve axonal marker, NF200 (red). (B). Images of Panx 1 immunostaining (red) and, Nissl staining (green) in the DRG; Scale bar = 50 μm. Enlarged images in lower panel are from the inset boxes of upper panel. Upper panel, scale bar = 100 μm. Lower panel, scale bar = 50 μm. [file 12974_2022_2603_MOESM2_ESM.tif]

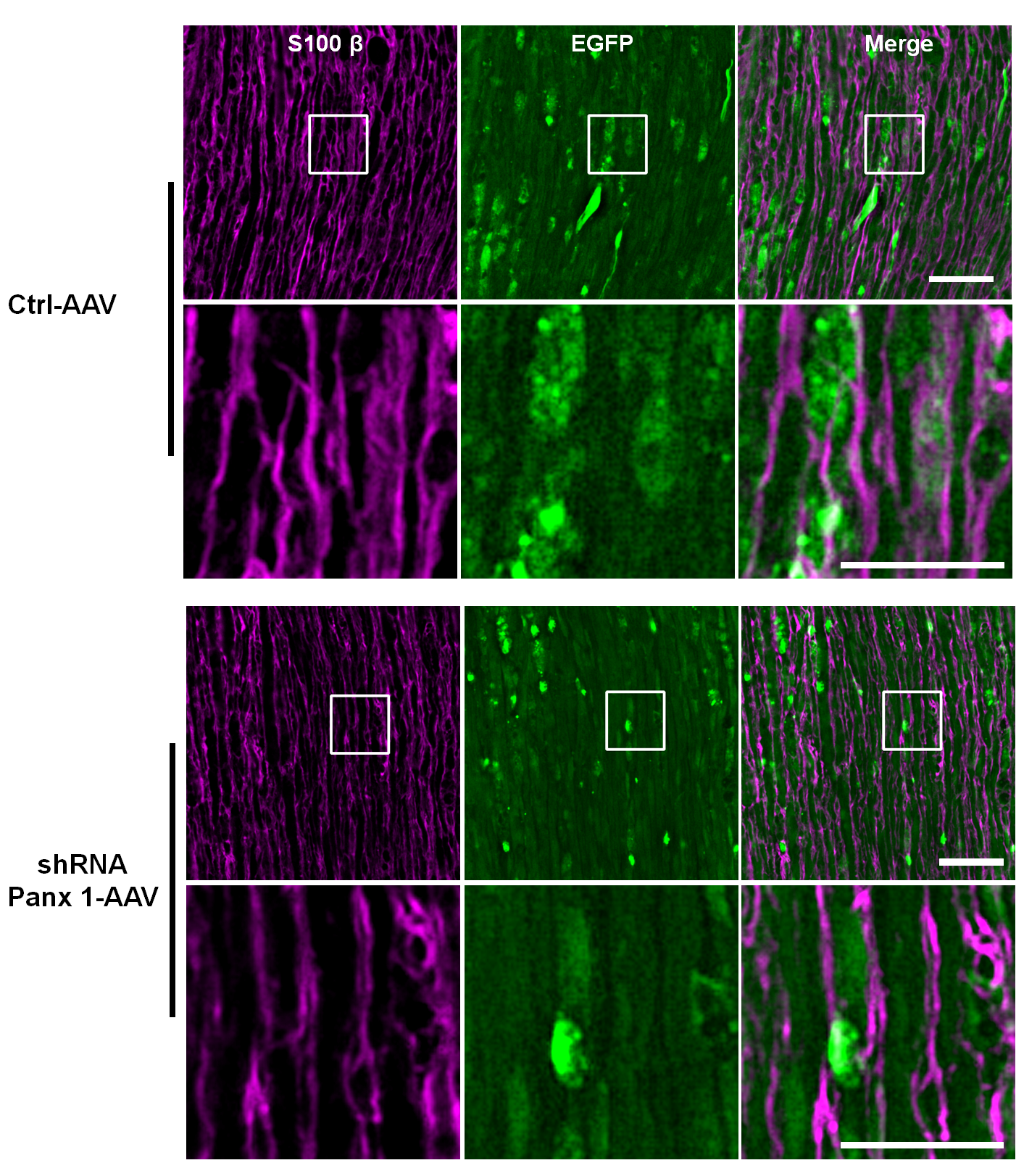

Supplement: Supplementary file 3 — Additional file 3: Figure S3. ShRNA Panx 1-AAV or Ctrl-AAV-labeled EGFP-positive cells have a sound co-localization with S100 β-labeled Schwann cells. Enlarged images in lower panel are from the inset boxes of upper panel. Upper panel, scale bar = 50 μm. Lower panel, scale bar = 25 μm. n = 3–4 mice/group. [file 12974_2022_2603_MOESM3_ESM.tif]

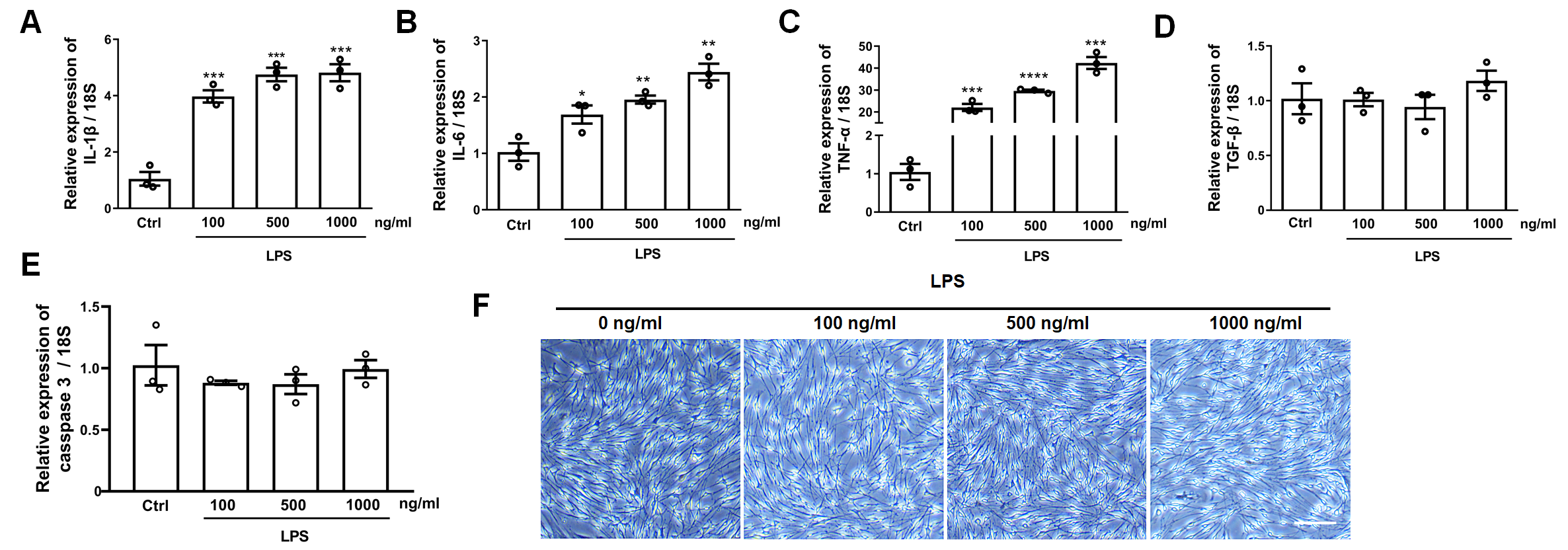

Supplement: Supplementary file 4 — Additional file 4: Figure S4. The responses of LPS-treated cultured Schwann cells. A-E. mRNA expression levels of the inflammatory factors, IL-1β, IL-6, TNF-α, and TGF-β (A-D), the apoptosis marker, caspase 3 (E) and in LPS-treated Schwann cells (0, 100, 500, and 1000 ng/ml, 24 h at 37℃). F. Cytomorphology of LPS-treated Schwann cells at different doses (0, 100, 500, and 1000 ng/ml) under a light microscope. All data are mean ± SEM. n = 3. The data were analyzed by one-way ANOVA followed by Tukey’s multiple comparison test. *p < 0.05, **p < 0.01, ***p < 0.001, vs. the control group. Scale bar = 100 μm. [file 12974_2022_2603_MOESM4_ESM.tif]

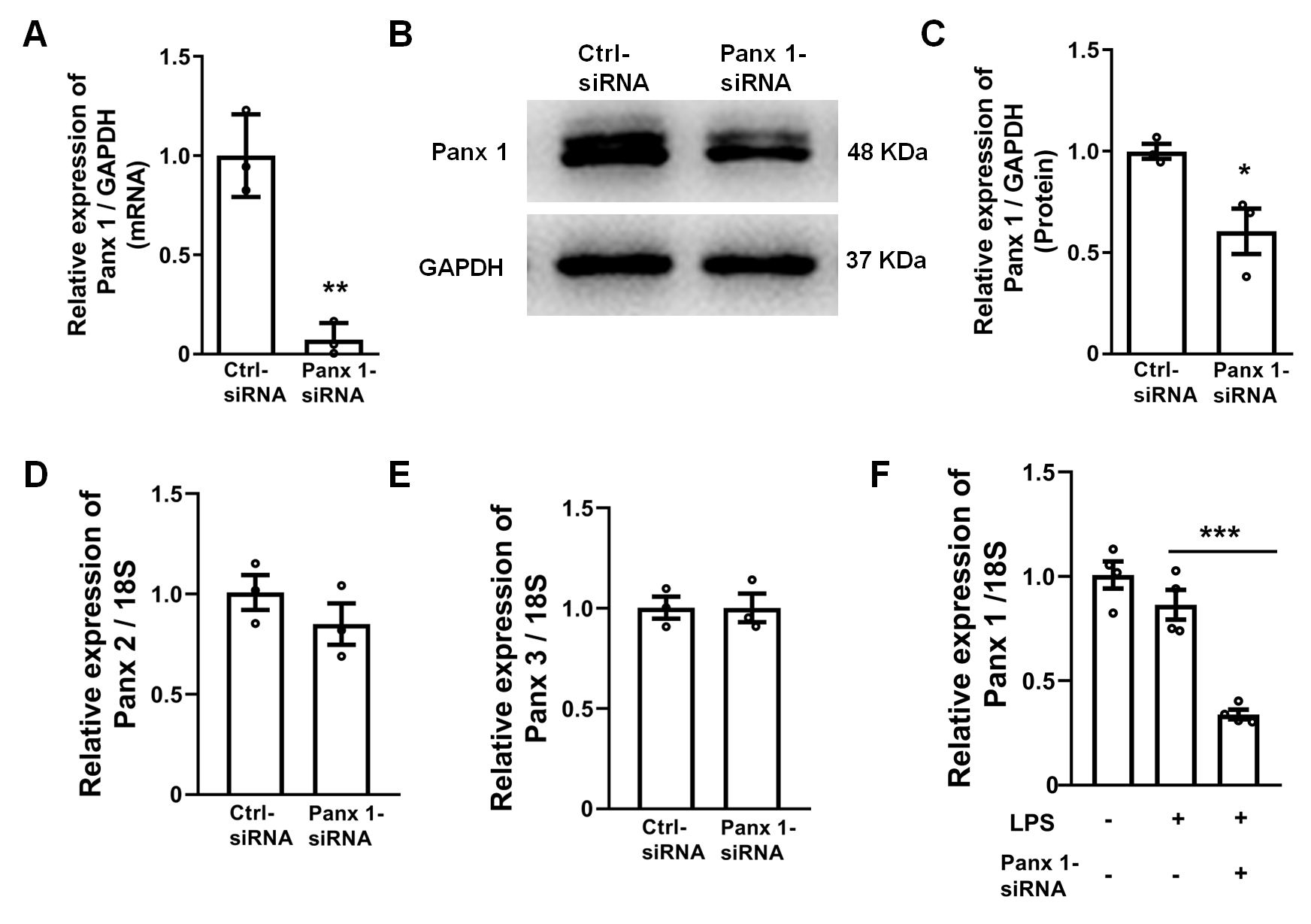

Supplement: Supplementary file 5 — Additional file 5: Figure S5. The effect of Panx 1-siRNA on the expression of Panx 1 in Schwann cells. A, D, and E. mRNA expression of Panx 1, Panx 2, and Panx 3 after treatment with Panx 1 siRNA. B-C. Gel images and quantification of Panx 1 protein levels after treatment with Panx 1 siRNA. F. Panx 1-specific siRNA decreased Panx 1 mRNA expression levels under LPS treatment in Schwann cells. All data are mean ± SEM. n = 3. Two groups in A and C were analyzed with Student’s t-test. The differences between the three groups in F were analyzed using one-way ANOVA following by Tukey’s multiple comparison test. *p < 0.05, **p < 0.01, vs. the control group. ***p < 0.001, vs. the LPS treatment alone group. [file 12974_2022_2603_MOESM5_ESM.tif]

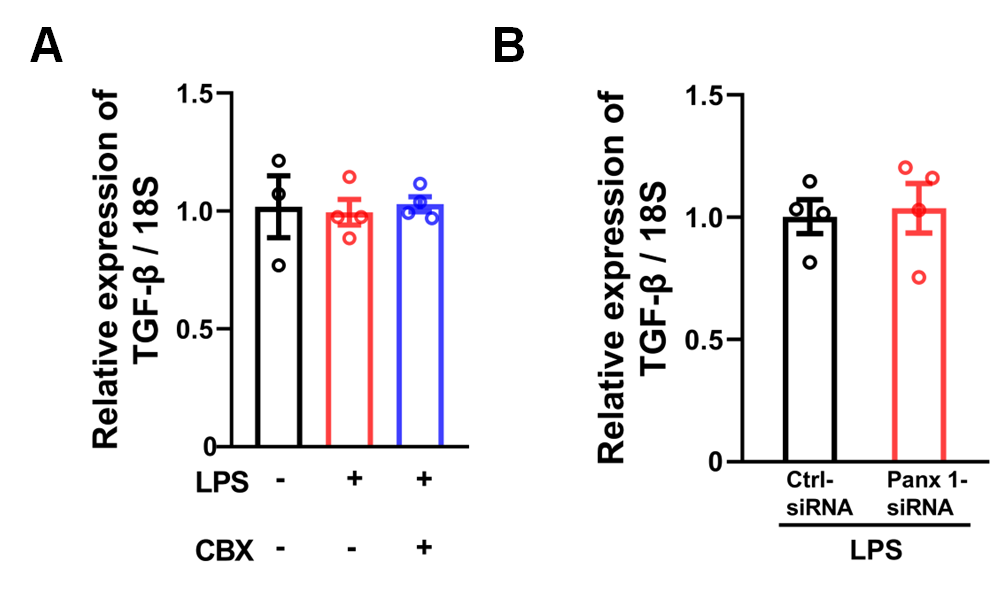

Supplement: Supplementary file 6 — Additional file 6: Figure S6. CBX and Panx 1-siRNA do not affect TGF-β mRNA expression in LPS-treated Schwann cells. A-B. Pretreatment with CBX (100 mM) for 2 h or Panx 1 siRNA for 36 h, qPCR was used to determine TGF-β mRNA expression levels in LPS-treated Schwann cells. All data are mean ± SEM. n = 4. [file 12974_2022_2603_MOESM6_ESM.tif]

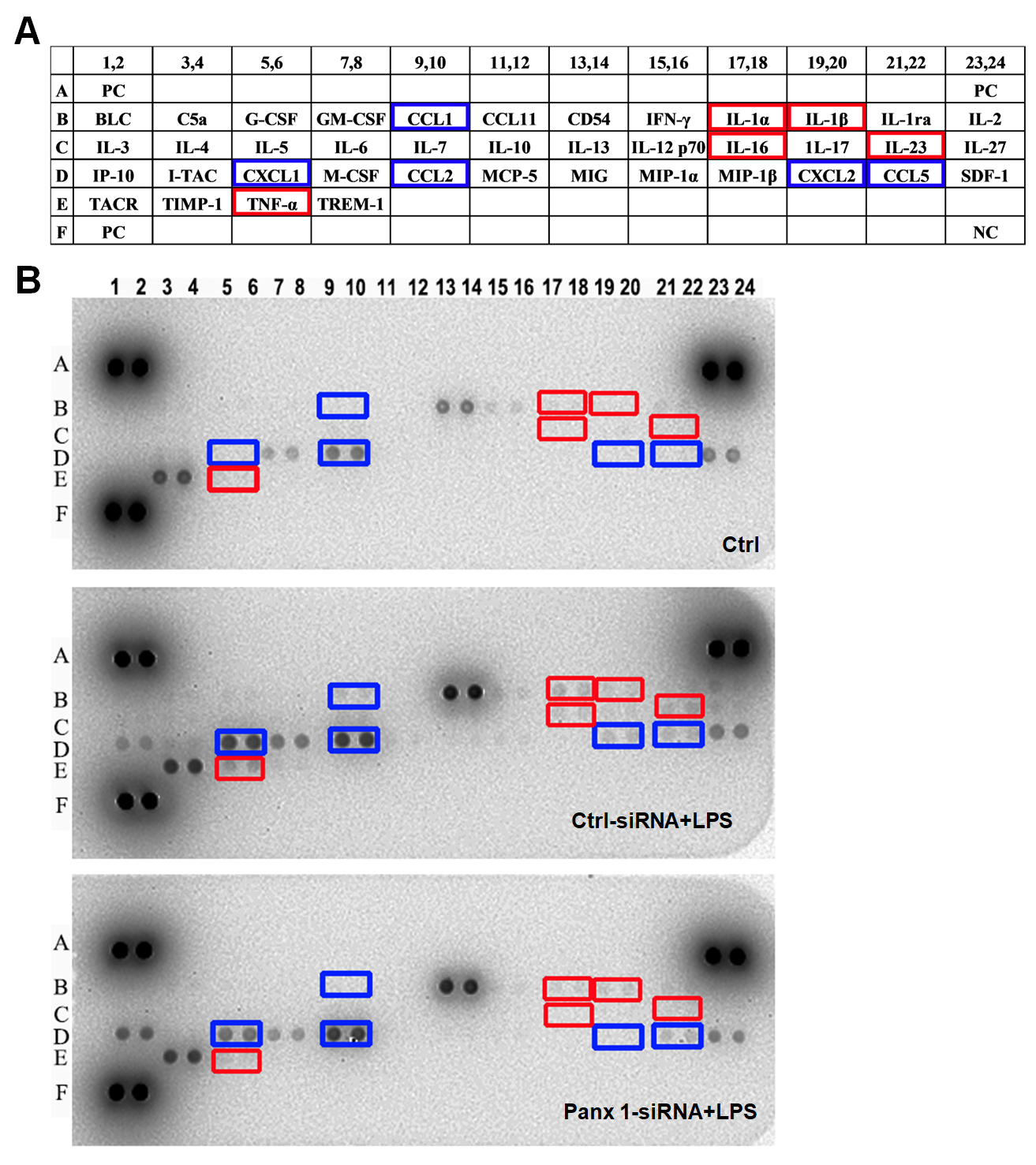

Supplement: Supplementary file 7 — Additional file 7: Figure S7. Cytokine array reveals Panx 1-dependent cytokines and chemokines in LPS-treated Schwann cells. A. Coordinates of the cytokine array containing 40 different cytokines and chemokines, including three positive control (PC) proteins. B. Array membranes of protein expression among Ctrl, Ctrl-siRNA, and Panx 1-siRNA groups in LPS-treated Schwann cells. [file 12974_2022_2603_MOESM7_ESM.tif]
